# Supplementary material for: FERN – a Java framework for stochastic simulation and evaluation of reaction networks
Source: BMC Bioinformatics. 2008 Aug 29;9:356. doi: 10.1186/1471-2105-9-356 (PMC2553347; doi:10.1186/1471-2105-9-356)
Supplement: Additional file 1 — FERN distribution, Version 1.3. This archive contains the FERN source code and binaries as well as documentation and example models in FernML and SBML. [file 1471-2105-9-356-S1.zip › fern/doc/javadoc/index-files/index-13.html]

N-Index


---


|  |  |  |
| --- | --- | --- |
| **Overview** \| Package \| Class \| Use \| **Tree** \| **Deprecated** \| **Index** \| **Help** | |  |
| **PREV LETTER**   **NEXT LETTER** | **FRAMES**    **NO FRAMES**     **All Classes** |  |


A B C D E F G H I J L M N O P R S T U V 

---


## **N**

**name** - Variable in class fern.network.AbstractNetworkImpl: Stores the network's identifier. **network** - Variable in class fern.analysis.AnalysisBase: Contains the network. **Network** - Interface in fern.network: The central interface of the whole framework. **NetworkChecker** - Class in fern.cytoscape: **NetworkChecker()** - Constructor for class fern.cytoscape.NetworkChecker: **NetworkChecker(String, Object, Object, String, String)** - Constructor for class fern.cytoscape.NetworkChecker: **NetworkChecker.EdgeClassifier** - Interface in fern.cytoscape: **NetworkChecker.EdgeClassifierByDirection** - Class in fern.cytoscape: **NetworkChecker.EdgeClassifierByDirection()** - Constructor for class fern.cytoscape.NetworkChecker.EdgeClassifierByDirection: **NetworkChecker.EdgeClassifierByIdentifier** - Class in fern.cytoscape: **NetworkChecker.EdgeClassifierByIdentifier()** - Constructor for class fern.cytoscape.NetworkChecker.EdgeClassifierByIdentifier: **NetworkChecker.NodeClassifier** - Interface in fern.cytoscape: **NetworkChecker.NodeClassifierByAnnotation**<T> - Class in fern.cytoscape: **NetworkChecker.NodeClassifierByAnnotation(String, T, T)** - Constructor for class fern.cytoscape.NetworkChecker.NodeClassifierByAnnotation: **NetworkChecker.NodeParameter** - Class in fern.cytoscape: **NetworkChecker.NodeParameter(String, String)** - Constructor for class fern.cytoscape.NetworkChecker.NodeParameter: **NetworkLoader** - Class in fern.network: **NetworkLoader()** - Constructor for class fern.network.NetworkLoader: **NetworkSearchAction** - Interface in fern.analysis: Implementing classes of `NetworkSearchAction` are able to control/watch searches in `AnalysisBase`. **NetworkSearchAction.NeighborType** - Enum in fern.analysis: Defines different types of neighborhoods in a `Network`. **NetworkTools** - Class in fern.tools: Contains various helper methods dealing with the `Network` interface. **NetworkTools()** - Constructor for class fern.tools.NetworkTools: **NodeChecker** - Interface in fern.analysis: Implementing classes can be used for a `NetworkSearchAction`s `checkReaction`, `checkSpecies`, if the information whether or not to visit the nodes is not accessible for the `NetworkSearchAction`. **NodeCheckerByAnnotation** - Class in fern.analysis: An instance of `NodeCheckerByAnnotation` can be used to control a search in `AnalysisBase` by a `NetworkSearchAction`. **NodeCheckerByAnnotation(String, String)** - Constructor for class fern.analysis.NodeCheckerByAnnotation: Creates the NodeChecker with the specified annotation field and value. **nodeType** - Variable in class fern.cytoscape.NetworkChecker: **nodeTypeReaction** - Variable in class fern.cytoscape.NetworkChecker: **nodeTypeSpecies** - Variable in class fern.cytoscape.NetworkChecker: **NumberTools** - Class in fern.tools: Contains some common methods to handle number related data structures. **NumberTools()** - Constructor for class fern.tools.NumberTools

---


|  |  |  |
| --- | --- | --- |
| **Overview** \| Package \| Class \| Use \| **Tree** \| **Deprecated** \| **Index** \| **Help** | |  |
| **PREV LETTER**   **NEXT LETTER** | **FRAMES**    **NO FRAMES**     **All Classes** |  |


A B C D E F G H I J L M N O P R S T U V 

---
